# Convention of Medical Teachers

**Published:** 1859-05

**Authors:** 


### CONVENTION OF MEDICAL TEACHERS.

This body assembled, in pursuance to a call, met at Louisville on Monday, May 1. We subjoin an abstract of its proceedings.

About twenty-one medical colleges, less than one-half probably, were represented.

Among those not represented may be noted the medical department of Yale College, all the schools of New York, Philadelphia, New Orleans, and the University of Nashville.

These, the largest institutions of the country, did not wish evidently to recognize any authority of the Convention, which sending a delegation might be construed as doing. Others, opposed to recognizing any action as binding, thought it still best to be represented. Among these was Rush Medical College.

The schools which expected to make capital out of the action of the Convention were fully represented. The report shows but imperfectly the spirit of the proceedings. It may be said, without danger of going too far, that a large part of the schools represented evinced a determination not to permit so changeable a body as the National Association to *prescribe* and *enforce* the terms on which they shall confer degrees. All not represented may be considered as either protesting against such an assumption, or as regarding the attempt as not of sufficient importance to deserve notice.

This abstract is unavoidably deferred to the next number.

---

In reply to some questions by the editor of the *Ohio Medical Journal*, we beg to state that, in the notice of M. Flouren's book, we only gave the conclusions of the author, without intending to support or dissent from them. The book in itself, and on account of its authority, seemed of sufficient importance in a physiological point of view, to justify a notice.

